# P-822. Invasive but Ineffective: Routine Mycoplasma testing for CAP: A Retrospective Study

**DOI:** 10.1093/ofid/ofaf695.1030

**Published:** 2026-01-11

**Authors:** R H E A BOHRA, R A J A E S W A R A N CHINNAMUTHU, H A R I N I V A A S SHANMUGAVEL, W A R R E N FERNANDES, S O P H E E NIRAULA, A D I T I LUITEL, S M R I T I DHAKAL, S U S H A I L I PRADHAN, A N N LIM, T A K U G O CHO

**Affiliations:** Saint Vincent Hospital, Worcester, MA; Saint Vincent Hospital, Worcester, MA; Saint Vincent Hospital, Worcester, MA; Saint Vincent Hospital, Worcester, MA; Memorial Sloan Kettering Cancer Center, MANHATTAN, New York; Albert Einstein College of Medicine, BRONX, New York; Chicago Public Schools, CHICAGO, Illinois; Care New England Health System, PAWTUCKET, Rhode Island; Saint Vincent Hospital, Worcester, MA; Saint Vincent Hospital, Worcester, MA

## Abstract

**Background:**

Community-acquired pneumonia (CAP) is the second most common cause of hospitalization in the United States and frequently involves atypical pathogens such as *Mycoplasma pneumoniae* (Mp). Current guidelines recommend empiric atypical coverage for CAP without necessitating specific Mp testing. Despite this, routine Mp serology testing (IgM) is frequently ordered. We conducted a retrospective study to assess whether routine Mp serologic testing impacts clinical management or outcomes in hospitalized CAP patients.

**Figure:**
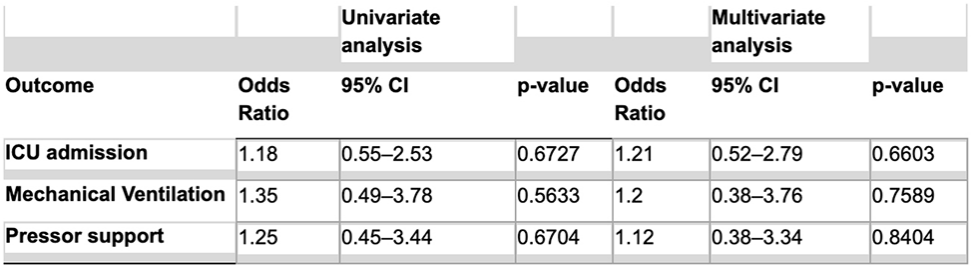
Univariate vs. Multivariate Analysis: Mycoplasma Serology and Critical Care Outcomes

**Figure:**
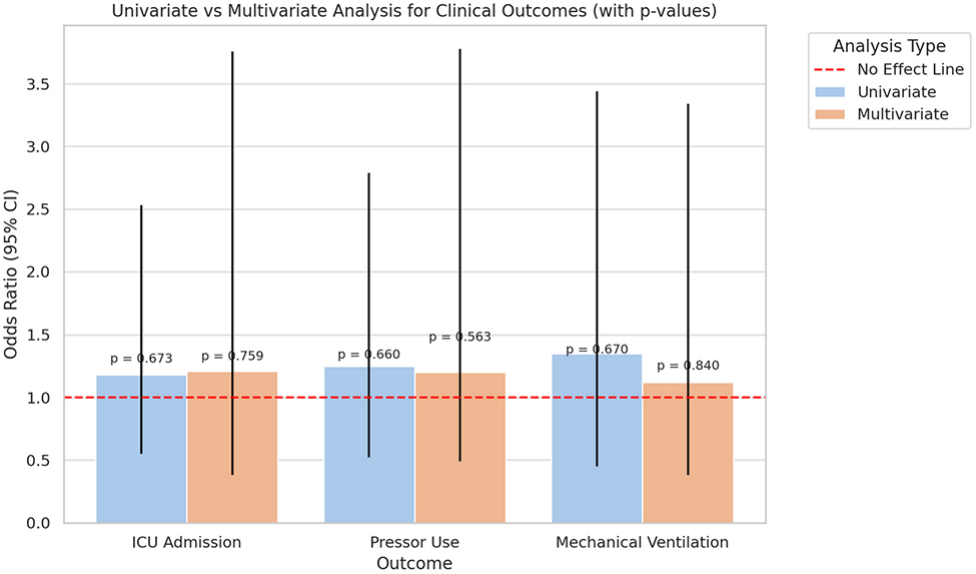
Univariate vs. Multivariate Logistic Regression for Critical Care Outcomes

**Methods:**

A retrospective chart review of patients >18 years, admitted to a community hospital from January 2023 to January 2024 with CAP was done. Patients were categorized based on whether Mp serology was performed. Data related to demographics, Charlson Comorbidity Index [CCI], Pneumonia Severity Index [PSI], smoking status, hospital length of stay, ICU admission, mechanical ventilation (MV) requirement and pressor use was collected. Outcomes were compared using logistic and linear regression models after adjusting for the covariates using Statistical Analysis Software® Version 9.4. The significance was set at 5%.

**Figure:**
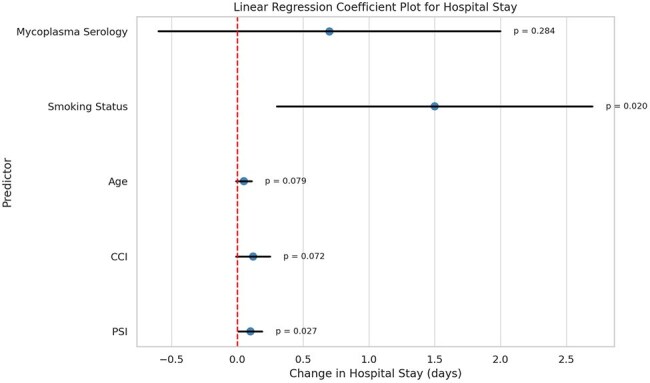
Predictors of Length of Stay: Multivariate Linear Regression Coefficients

**Results:**

Serology testing was not significantly associated with differences in hospital length of stay (mean 7.0 vs. 6.3 days; *p* = 0.2841). ICU admission rates were similar between groups, with a non-significant trend toward increased ICU use among those tested (OR = 1.21; *p* = 0.6603). Likewise, the need for pressor support was slightly higher in the serology-tested group, though this did not reach statistical significance (OR = 1.20; *p* = 0.7589). In total, 49 Mycoplasma serology tests were performed, contributing approximately $5,000 in additional direct healthcare costs.

**Conclusion:**

Routine *Mycoplasma pneumoniae* testing in hospitalized patients with CAP was not associated with improved clinical outcomes. Instead, the use of serologic testing in low-suspicion cases appeared to increase resource utilization without a clear diagnostic or therapeutic benefit. Given the limited value in such contexts, these findings support a more judicious application of *Mycoplasma* serology testing, emphasizing clinical judgment to guide diagnostic decisions in support of high-value, cost-effective care.

**Disclosures:**

All Authors: No reported disclosures

